# Supplementary material for: A tightly clustered hepatitis E virus genotype 1a is associated with endemic and outbreak infections in Bangladesh
Source: PLoS One. 2021 Jul 22;16(7):e0255054. doi: 10.1371/journal.pone.0255054 (PMC8297744; doi:10.1371/journal.pone.0255054)
Supplement: S1 File — (DOCX) [file pone.0255054.s001.docx]

S1 File: List of primers used for whole genome amplification in this study

| **Primer set** | **Primer name** | **Orientation** | **Primer sequence (5' - 3')** | **Sequence position*** |
| --- | --- | --- | --- | --- |
|  | HEV-5end-S1 | sense | **GGCAGACCACGTATGTGGTCGATGC** | 1 - 25 |
|  | HEV-5end-A1 | 1st antisense | **CGCAGCGCGGGAACGCCGGC** | 412-394 |
| 5’end | HEV-5end-A2 | 2nd antisense | **AGGGCGATGCCTGTCTCGGCGG** | 493-472 |
|  | HEV-S1-A2 | 3rd antisense | **GTCATRCCATGGCGGAACAT** | 556-537 |
| **1** | HEV-S2-S1 | 1st sense | **CCTAATGTGGTCCACCGCTG** | 303 - 322 |
|  | HEV-S2-S2 | 1st antisense | **GACCTCGGTRGACCGGGGGTA** | 866 - 846 |
|  | HEV-S2-A1 | 2nd sense | **GTTCAGCGYTGGTATACTGC** | 348 - 367 |
|  | HEV-S2-A2 | 2nd antisense | **GATGGCTCCGGGGCDGCCGT** | 824 - 804 |
| **2** | HEV-S3-S1 | 1st sense | **GGCAGGCGYGTTGTGGTGAC** | 646 - 665 |
|  | HEV-S3-S2 | 1st antisense | **CATCCCCTTRGATATAGCCTG** | 1190 - 1170 |
|  | HEV-S3-A1 | 2nd sense | **GAGGGTGAYACYAGTGCTGG** | 669 - 688 |
|  | HEV-S3-A2 | 2nd antisense | **GCGGCRGTRATGACAGCTGT** | 1132 - 1113 |
| **3** | HEV-S4-S1 | 1st sense | **ACCTCATGCTCCACTAAGTC** | 915 - 934 |
|  | HEV-S4-S2 | 1st antisense | **TCCTGRCCMAGCCACTTCAT** | 1444 - 1425 |
|  | HEV-S4-A1 | 2nd sense | **GTCCCTGCCCATATYTGGGA** | 948 - 967 |
|  | HEV-S4-A2 | 2nd antisense | **CARTGGCAGGGGGCCGACTC** | 1381 - 1362 |
| **4** | HEV-S5-S1 | 1st sense | **GGCTCTTYGAGAAGTCCGGCCG** | 1247 - 1267 |
|  | HEV-S5-S2 | 1st antisense | **GTCTCRCARTCGATCCGCCC** | 1699 - 1680 |
|  | HEV-S5-A1 | 2nd sense | **GCYCAGTGYAGGCGCTGGCT** | 1299 - 1318 |
|  | HEV-S5-A2 | 2nd antisense | **TTTACTGTGGCGGTCAGCCG** | 1663 - 1644 |
| **5** | HEV-S6-S1 | 1st sense | **GCCTATGAGGGGTCYGATGT** | 1506 - 1525 |
|  | HEV-S6-S2 | 1st antisense | **TTAGCCGACTCCCARACATG** | 2080 - 2061 |
|  | HEV-S6-A1 | 2nd sense | **AGTGAYATMTCTGGGTCCTA** | 1548 - 1567 |
|  | HEV-S6-A2 | 2nd antisense | **AGYCCCTCAGGRTGGAACCA** | 2029 - 2010 |
| **6** | HEV-S7-S1 | 1st sense | **AGTCAGAGCACTATGGCHGC** | 1794 - 1813 |
|  | HEV-S7-S2 | 1st antisense | **CGACTCRAACAGCGAGCCGGC** | 2399 - 2379 |
|  | HEV-S7-A1 | 2nd sense | **CGCTATGTYGCTGCCGGGCT** | 1863 - 1882 |
|  | HEV-S7-A2 | 2nd antisense | **CCRYCTGGTGGGTTATGGCC** | 2328 - 2309 |
| **7** | HEV-S8-S1 | 1st sense | **GAGAGCACACTYTACACCCG** | 2094 - 2113 |
|  | HEV-S8-S2 | 1st antisense | **TATATGCCRGTCCCGAGGAG** | 2677 - 2658 |
|  | HEV-S8-A1 | 2nd sense | **ACTTGGTCGGARGTTGATGC** | 2115 - 2134 |
|  | HEV-S8-A2 | 2nd antisense | **CTTTGGGTTATGTTCCAACCTATA** | 2600 - 2577 |
| **8** | HEV-S9-S1 | 1st sense | **TAATGTTGACCAYCGCCCTGG** | 2426 - 2446 |
|  | HEV-S9-S2 | 1st antisense | **GGCACACCTGCRGTYAACTG** | 2935 - 2916 |
|  | HEV-S9-A1 | 2nd sense | **GATGCTGCCTCTTTTGTGATG** | 2495 - 2514 |
|  | HEV-S9-A2 | 2nd antisense | **CCCGGCCGACRTCTGTGGC** | 2874 - 2856 |
| **9** | HEV-S10-S1 | 1st sense | **TTTGACGCCTGGGAGCGGAA** | 2710 - 2719 |
|  | HEV-S10-S2 | 1st antisense | **TGATGGCGGGRACRAGCCC** | 3222 - 3204 |
|  | HEV-S10-A1 | 2nd sense | **GCCARATGGTTYGAGGCCAA** | 2762 - 2781 |
|  | HEV-S10-A2 | 2nd antisense | **ACGGTGGCGGCCCGCTGCAT** | 3151 - 3132 |
| **10** | HEV-S11-S1 | 1st sense | **GGACGTTGTYGTGGTYCCGAC** | 2975 - 2995 |
|  | HEV-S11-S2 | 1st antisense | **CCYGGTGCGTCAATGATGAC** | 3568 - 3549 |
|  | HEV-S11-A1 | 2nd sense | **TGCGYAAYGCCTGGCGCCG** | 3004 - 3022 |
|  | HEV-S11-A2 | 2nd antisense | **ACTTCTCAGTGTGGCGCGTC** | 3546 - 3527 |
| **11** | HEV-S12-S1 | 1st sense | **GATYCAGACCACTAGYCGGGT** | 3311 - 3331 |
|  | HEV-S12-S2 | 1st antisense | **AAATGTYARGACACTATCACAG** | 3855 - 3836 |
|  | HEV-S12-A1 | 2nd sense | **GAARYTAGTGTTCACCCAGGC** | 3371 - 3391 |
|  | HEV-S12-A2 | 2nd antisense | **AGGTAGAGRAGGCCCTGTTC** | 3823 - 3804 |
| **12** | HEV-S13-S1 | 1st sense | **AATGTYGACACCYTGGCTGCC** | 3681 - 3701 |
|  | HEV-S13-S2 | 1st antisense | **TCGAGCTCAAGGACGGCGGAGCC** | 4111 - 4091 |
|  | HEV-S13-A1 | 2nd sense | **TTYCCNCCGTCTTGCCAGATTAG** | 3704 - 3724 |
|  | HEV-S13-A2 | 2nd antisense | **TGGCCCTTCTCRACCATGGC** | 4084 - 4065 |
| **13** | HEV-S14-S1 | 1st sense | **CGAGNCAGCGCAAGGCYGT** | 3901 - 3919 |
|  | HEV-S14-S2 | 1st antisense | **TCCWCCATAATAGCACACTC** | 4450 - 4431 |
|  | HEV-S14-A1 | 2nd sense | **GTYCGCGACTCTCTCGCCCG** | 3984 - 4003 |
|  | HEV-S14-A2 | 2nd antisense | **CAGARAARTCATTCTCAAACACCAT** | 4392 - 4368 |
| **14** | HEV-S15-S1 | 1st sense | **CATGGYAAAGTGGGYCAGGG** | 4191 - 4210 |
|  | HEV-S15-S2 | 1st antisense | **ACGRTACTCACTGCAAAGCAC** | 4688 - 4668 |
|  | HEV-S15-A1 | 2nd sense | **TTCCGYGCTATTGAGAAGGC** | 4257 - 4276 |
|  | HEV-S15-A2 | 2nd antisense | **GAATCATCACCTTTRAARGC** | 4664 - 4644 |
| **15** | HEV-S16-S1 | 1st sense | **GATYYTGCAGGCCCCGAAGGA** | 4505 - 4525 |
|  | HEV-S16-S2 | 1st antisense | **ATRCCAATCAGGTTATGAAC** | 4993 - 4974 |
|  | HEV-S16-A1 | 2nd sense | **AAACACTCCGGTGAGCCYGGCA** | 4551 - 4570 |
|  | HEV-S16-A2 | 2nd antisense | **GGGGARACCCCATARACACG** | 4966 - 4947 |
| **16** | HEV-S17-S1 | 1st sense | **GCAGGTGTTGTGGTGGCCCC** | 4770 - 4789 |
|  | HEV-S17-S2 | 1st antisense | **GGCGAAGGGGTTGGTTGGATG** | 5313 - 5293 |
|  | HEV-S17-A1 | 2nd sense | **ACYGAGAAGAATTGGGGCCC** | 4833 - 4852 |
|  | HEV-S17-A2 | 2nd antisense | **TAGGGGATTGCGAAGGGCTGAG** | 5288 - 5267 |
| **17** | HEV-S18-S1 | 1st sense | **GGGTGGAATGAATAACATGTCT** | 5077 - 5098 |
|  | HEV-S18-S2 | 1st antisense | **CAACCCGGTACTGGGCATAA** | 5686 - 5667 |
|  | HEV-S18-A1 | 2nd sense | **ATGCGCCCTCGGCCTATTTTG** | 5125 - 5145 |
|  | HEV-S18-A2 | 2nd antisense | **ATGRGTATTGGTRCCGTCCTG** | 5643 - 5623 |
| **18** | HEV-S19-S1 | 1st sense | **TTGGCGTGACCAGGCCCAGCG** | 5382 - 5402 |
|  | HEV-S19-S2 | 1st antisense | **GCCATGTATRCARAGCATRACAAG** | 5970 - 5947 |
|  | HEV-S19-A1 | 2nd sense | **GTAGACCTRCCACAGCTGGG** | 5423 - 5442 |
|  | HEV-S19-A2 | 2nd antisense | **GCYTCCTCCTCMGCCACCCC** | 5936 - 5917 |
| **19** | HEV-S20-S1 | 1st sense | **ACCACCACCCCGACGTCCGT** | 5770 - 5789 |
|  | HEV-S20-S2 | 1st antisense | **TGTCAGCAAGGTTRAAMAGGGT** | 6256 - 6236 |
|  | HEV-S20-A1 | 2nd sense | **AATTCAATAACYTCGACKGATGT** | 5803 - 5822 |
|  | HEV-S20-A2 | 2nd antisense | **GCTATCCCGCGGCCGATCTC** | 6230 - 6211 |
| **20** | HEV-S21-S1 | 1st sense | **CGCAACCTYACCCCYGGTAA** | 6049 - 6068 |
|  | HEV-S21-S2 | 1st antisense | **GGACTGGTCATACTCGGCAGC** | 6573 - 6553 |
|  | HEV-S21-A1 | 2nd sense | **GTCTCCCGTTAYTCCAGCAC** | 6082 - 6101 |
|  | HEV-S21-A2 | 2nd antisense | **AGAGARAGCCAAAGCACATC** | 6548 - 6529 |
| **21** | HEV-S22-S1 | 1st sense | **ACAGAATTGATTTCGTCGGC** | 6277 - 6296 |
|  | HEV-S22-S2 | 1st antisense | **TTRTAATTATARGGGTACCC** | 6801 - 6788 |
|  | HEV-S22-A1 | 2nd sense | **CTGTRGAGAATGCTCAGCAGG** | 6368 - 6388 |
|  | HEV-S22-A2 | 2nd antisense | **CGRAGCGGCAGGACAAAGAA** | 6749 - 6730 |
| **22** | HEV-S23-S1 | 1st sense | **TCYGACTCTGTGACCYTGGT** | 6607 - 6626 |
|  | HEV-S23-S2 | 1st antisense | **CAAGCAAATAAACTATAACTCCCG** | 7119 - 7096 |
|  | HEV-S23-A1 | 2nd sense | **ACCGGCGCGCAGGCCGTTGC** | 6637 - 6655 |
|  | HEV-S23-A2 | 2nd antisense | **GTTTTACCCACCTTCATCTTAAG** | 7094 - 7071 |
| **3’end** | HEV-3end-S1 | sense | **CTAGCCTGGGTGCTGGCCCCGTCT** | 7015 - 7038 |
|  | HEV-3end-A1 | antisense | **TTTTCAGGGAGCGCGAAACGC** | 7230 - 7210 |
|  |  |  |  |  |

* Sequence position is based on HEV reference sequence E13-Ban10 (GenBank accession no AB720035.1)
