# Supplementary material for: A tightly clustered hepatitis E virus genotype 1a is associated with endemic and outbreak infections in Bangladesh
Source: PLoS One. 2021 Jul 22;16(7):e0255054. doi: 10.1371/journal.pone.0255054 (PMC8297744; doi:10.1371/journal.pone.0255054)
Supplement: S2 File — (DOCX) [file pone.0255054.s002.docx]

S2 File: Accession number, genotype and subtype, and country of origin for 109 Hepatitis E virus used as representative reference strain

| **No.** | **Accession Number** | **Genotype** | **Origin country** |
| --- | --- | --- | --- |
| 1 | JQ655734.1 | 1 | China |
| 2 | NC001434 | 1 | China |
| 3 | AB720034.1 | 1a | Bangladesh |
| 4 | AB720035.1 | 1a | Bangladesh |
| 5 | AF051830 | 1a | Nepal |
| 6 | AF076239 | 1a | India |
| 7 | AF185822 | 1a | Pakistan |
| 8 | AF459438 | 1a | India |
| 9 | D10330 | 1a | Myanmar |
| 10 | DQ459342 | 1a | India |
| 11 | FJ457024 | 1a | India |
| 12 | JF443717.1 | 1a | India |
| 13 | JF443718.1 | 1a | India |
| 14 | JF443719.1 | 1a | India |
| 15 | JF443720 | 1a | India |
| 16 | JF443721 | 1a | India |
| 17 | JF443722 | 1a | India |
| 18 | JF443723 | 1a | India |
| 19 | JF443724 | 1a | India |
| 20 | JF443725 | 1a | India |
| 21 | JF443726 | 1a | India |
| 22 | M73218 | 1a | Burma |
| 23 | X99441 | 1a | India |
| 24 | AF444002 | 1b | Pakistan |
| 25 | D11092 | 1b | China |
| 26 | D11093 | 1b | China |
| 27 | L08816 | 1b | China |
| 28 | L25547 | 1b | China |
| 29 | M80581 | 1b | Pakistan |
| 30 | M94177 | 1b | China |
| 31 | X98292 | 1c | India |
| 32 | AY230202 | 1d | Morocco |
| 33 | AY204877 | 1e | Chad |
| 34 | M74506 | 2a | Mexico |
| 35 | AB369689.1 | 3 | Japan |
| 36 | EU723513 | 3 | Spain |
| 37 | JQ013791 | 3 | France |
| 38 | EU360977 | 3 | Sweden |
| 39 | AB290312 | 3 | Mongolia |
| 40 | AB290313 | 3 | Mongolia |
| 41 | JQ953664 | 3 | France |
| 42 | KJ013415 | 3? | China |
| 43 | AB074918 | 3a | Japan |
| 44 | AB074920 | 3a | Japan |
| 45 | AB089824 | 3a | Japan |
| 46 | AF060668 | 3a | USA |
| 47 | AF082843 | 3a | USA |
| 48 | AB091394 | 3b | Japan |
| 49 | AB189072 | 3b | Japan |
| 50 | AP003430 | 3b | Japan |
| 51 | FJ705359 | 3c | Germany |
| 52 | AB248520 | 3e | Japan |
| 53 | AB248521 | 3e | Japan |
| 54 | AB369687.1 | 3f | Japan |
| 55 | AF455784 | 3g | Kyrgyzstan |
| 56 | JQ013794 | 3h | France |
| 57 | FJ998008 | 3i | Germany |
| 58 | AY115488 | 3j | Canada |
| 59 | FJ906895 | 3 | China |
| 60 | AB220974.1 | 4 | Japan |
| 61 | AB369688.1 | 4 | Japan |
| 62 | AB091395. | 4 | Japan |
| 63 | AB193176. | 4 | Japan |
| 64 | AB193177. | 4 | Japan |
| 65 | AB197673.1 | 4 | Japan |
| 66 | AB197674.1 | 4 | Japan |
| 67 | AB220971.1 | 4 | Japan |
| 68 | AB220972.1 | 4 | Japan |
| 69 | AB220973.1 | 4 | Japan |
| 70 | AB220975.1 | 4 | Japan |
| 71 | AB220976.1 | 4 | Japan |
| 72 | AB220977.1 | 4 | Japan |
| 73 | AB220978.1 | 4 | Japan |
| 74 | AB220979.1 | 4 | Japan |
| 75 | AB253420.1 | 4 | Japan |
| 76 | AB291959.1 | 4 | Japan |
| 77 | AB291964.1 | 4 | Japan |
| 78 | AB291965.1 | 4 | Japan |
| 79 | AB291966.1 | 4 | Japan |
| 80 | AB291967.1 | 4 | Japan |
| 81 | AB291968.1 | 4 | Japan |
| 82 | AB369690.1 | 4 | Japan |
| 83 | AB480825.1 | 4 | Japan |
| 84 | AB521805.1 | 4 | Japan |
| 85 | AB521806.1 | 4 | Japan |
| 86 | AB698654.1 | 4 | Japan |
| 87 | JQ655733.1 | 4 | Japan |
| 88 | JQ655735.1 | 4 | China |
| 89 | JQ655736.1 | 4 | China |
| 90 | JQ740781.1 | 4 | China |
| 91 | KC163335.1 | 4 | China |
| 92 | DQ279091 | 4b | China |
| 93 | AB074915.3 | 4c | Japan |
| 94 | AB074917.3 | 4c | Japan |
| 95 | AB080575 | 4c | Japan |
| 96 | AB097812 | 4c | Japan |
| 97 | AB099347 | 4c | Japan |
| 98 | AB161717 | 4c | Japan |
| 99 | AJ272108 | 4d | China |
| 100 | AY723745 | 4e | India |
| 101 | AB220974 | 4f | Japan |
| 102 | AB108537 | 4g | China |
| 103 | GU119961 | 4h | China |
| 104 | DQ450072 | 4i | China |
| 105 | AB573435 | 5a | Japan |
| 106 | AB856243 | 6 | Japan |
| 107 | AB602441 | 6a | Japan |
| 108 | KJ496144 | 7 | United Arab Emirates |
| 109 | KJ496143 | 7a | United Arab Emirates |
