# Supplementary material for: A tightly clustered hepatitis E virus genotype 1a is associated with endemic and outbreak infections in Bangladesh
Source: PLoS One. 2021 Jul 22;16(7):e0255054. doi: 10.1371/journal.pone.0255054 (PMC8297744; doi:10.1371/journal.pone.0255054)
Supplement: S3 File — (DOCX) [file pone.0255054.s003.docx]

S3 File: Sociodemographic characteristics, HEV RNA positivity and HEV WGS information 92 patient samples analyzed in this study.

| Variable | | | 2013-2015 | 2013 - 2014 | 2014 - 2015 |
| --- | --- | --- | --- | --- | --- |
|  |  |  | No (%) | No (%) | No (%) |
| Sample collected | | | 92 (100) | 44 (47.8) | 48 (52.2) |
| Age (median; IQR) Yrs. | | | 25; 21-30 | 24.5; 20-31 | 25.5; 22-30 |
| Gender | | |  |  |  |
|  | Male | | 39 (42.3) | 18 (40.9) | 21 (43.7) |
|  | Female | | 53 (57.6) | 26 (59.0) | 27 (56.2) |
|  |  | Non pregnant | 29 (54.7) | 16 (61.5) | 13 (48.1) |
|  |  | Pregnant | 24 (45.3) | 10 (38.4) | 14 (51.8) |
| HEV RNA positive | | | 70 (76.1) | 31 (72.1) | 39 (79.6) |
| HEV partial sequence | | | 70 (76.1) | 31 (72.1) | 39 (79.6) |
| 286 bp ORF 2-3 region | | | 38 (54.2) | 19 (43.1) | 19 (39.5) |
| HEV WGS | | | 21 (22.8) | 13 (29.5) | 8 (16.6) |

IQR: inter quartile range
