# Supplementary material for: A tightly clustered hepatitis E virus genotype 1a is associated with endemic and outbreak infections in Bangladesh
Source: PLoS One. 2021 Jul 22;16(7):e0255054. doi: 10.1371/journal.pone.0255054 (PMC8297744; doi:10.1371/journal.pone.0255054)
Supplement: S4 File — Among 92 samples, 70 were qPCR positive (column 3), qPCR negative samples are presented in transparent rows. A total of 825 fragments (column 28) could be amplified and sequenced (dark block) (range 1–24). Whole genome sequence could be obtained for 21 HEV viral strain. Fragment 20 (nucleotide sequence 5822 to 6211) contains the ORF 2–3 overlapping section could be sequence in 38 (21+ 17) strain. (DOCX) [file pone.0255054.s004.docx]

S4 File: Schematic presentation of the sequencing of 92 HEV IgM ELISA positive samples. Among 92 samples, 70 were qPCR positive (column 3), qPCR negative samples are presented in transparent rows. A total of 825 fragments (last column) could be amplified and sequenced (presented as dark blocks) (range 1-24). Whole genome sequence could be obtained for 21 HEV strain. Fragment 20 (S20; nucleotide sequence 5822 to 6211) contains the ORF 2-3 overlapping section could be sequence in 38 (21+ 17) strain. Light shade blocks represents the qPCR positive samples where PCR amplification and sequencing was unsuccessful.

| **SL no** | **Sample ID** | **qPCR** | **5' region** | **S02 (367-804)** | **S03 (688-1113)** | **S04 (967-1362)** | **S05 (1318-1644)** | **S06 (1567-2010)** | **S07 (1882-2309)** | **S08 (2134-2577)** | **S09 (2514-2856)** | **S10 (2781-3132)** | **S11 (3022-3527)** | **S12 (3391-3804)** | **S13 (3724-4065)** | **S14 (4003-4368)** | **S15 (4276-4644)** | **S16 (4570-4947)** | **S17 (4852-5267)** | **S18 (5145-5623)** | **S19 (5442-5917)** | **S20 (5822-6211)** | **S21 (6101-6529)** | **S22 (6388-6730)** | **S23 (6655-7071)** | **3' region** | **Fragment**  **N=825** |
| --- | --- | --- | --- | --- | --- | --- | --- | --- | --- | --- | --- | --- | --- | --- | --- | --- | --- | --- | --- | --- | --- | --- | --- | --- | --- | --- | --- |
|  |  |  |  |  |  |  |  |  |  |  |  |  |  |  |  |  |  |  |  |  |  |  |  |  |  |  |  |
| **1** | **BDHEVF001** | **(-)** |  |  |  |  |  |  |  |  |  |  |  |  |  |  |  |  |  |  |  |  |  |  |  |  | **0** |
| **2** | **BDHEVF003** | **(-)** |  |  |  |  |  |  |  |  |  |  |  |  |  |  |  |  |  |  |  |  |  |  |  |  | **0** |
| **3** | **BDHEVF004** | **(+)** |  |  |  |  |  |  |  |  |  |  |  |  |  |  |  |  |  |  |  |  |  |  |  |  | **24** |
| **4** | **BDHEVF011** | **(-)** |  |  |  |  |  |  |  |  |  |  |  |  |  |  |  |  |  |  |  |  |  |  |  |  | **1** |
| **5** | **BDHEVF013** | **(+)** |  |  |  |  |  |  |  |  |  |  |  |  |  |  |  |  |  |  |  |  |  |  |  |  | **10** |
| **6** | **BDHEVF014** | **(-)** |  |  |  |  |  |  |  |  |  |  |  |  |  |  |  |  |  |  |  |  |  |  |  |  | **0** |
| **7** | **BDHEVF020** | **(+)** |  |  |  |  |  |  |  |  |  |  |  |  |  |  |  |  |  |  |  |  |  |  |  |  | **14** |
| **8** | **BDHEVF021** | **(+)** |  |  |  |  |  |  |  |  |  |  |  |  |  |  |  |  |  |  |  |  |  |  |  |  | **1** |
| **9** | **BDHEVF023** | **(+)** |  |  |  |  |  |  |  |  |  |  |  |  |  |  |  |  |  |  |  |  |  |  |  |  | **1** |
| **10** | **BDHEVF025** | **(+)** |  |  |  |  |  |  |  |  |  |  |  |  |  |  |  |  |  |  |  |  |  |  |  |  | **11** |
| **11** | **BDHEVF027** | **(+)** |  |  |  |  |  |  |  |  |  |  |  |  |  |  |  |  |  |  |  |  |  |  |  |  | **12** |
| **12** | **BDHEVF029** | **(+)** |  |  |  |  |  |  |  |  |  |  |  |  |  |  |  |  |  |  |  |  |  |  |  |  | **2** |
| **13** | **BDHEVF031** | **(+)** |  |  |  |  |  |  |  |  |  |  |  |  |  |  |  |  |  |  |  |  |  |  |  |  | **4** |
| **14** | **BDHEVF032** | **(+)** |  |  |  |  |  |  |  |  |  |  |  |  |  |  |  |  |  |  |  |  |  |  |  |  | **5** |
| **15** | **BDHEVF033** | **(+)** |  |  |  |  |  |  |  |  |  |  |  |  |  |  |  |  |  |  |  |  |  |  |  |  | **4** |
| **16** | **BDHEVF174** | **(-)** |  |  |  |  |  |  |  |  |  |  |  |  |  |  |  |  |  |  |  |  |  |  |  |  | **2** |
| **17** | **BDHEVF122** | **(+)** |  |  |  |  |  |  |  |  |  |  |  |  |  |  |  |  |  |  |  |  |  |  |  |  | **7** |
| **18** | **BDHEVF074** | **(+)** |  |  |  |  |  |  |  |  |  |  |  |  |  |  |  |  |  |  |  |  |  |  |  |  | **1** |
| **19** | **BDHEVF034** | **(+)** |  |  |  |  |  |  |  |  |  |  |  |  |  |  |  |  |  |  |  |  |  |  |  |  | **24** |
| **20** | **BDHEVF053** | **(+)** |  |  |  |  |  |  |  |  |  |  |  |  |  |  |  |  |  |  |  |  |  |  |  |  | **12** |
| **21** | **BDHEVFP02** | **(+)** |  |  |  |  |  |  |  |  |  |  |  |  |  |  |  |  |  |  |  |  |  |  |  |  | **2** |
| **22** | **BDHEVFP03** | **(+)** |  |  |  |  |  |  |  |  |  |  |  |  |  |  |  |  |  |  |  |  |  |  |  |  | **2** |
| **23** | **BDHEVFP06** | **(-)** |  |  |  |  |  |  |  |  |  |  |  |  |  |  |  |  |  |  |  |  |  |  |  |  | **0** |
| **24** | **BDHEVFP07** | **(-)** |  |  |  |  |  |  |  |  |  |  |  |  |  |  |  |  |  |  |  |  |  |  |  |  | **0** |
| **25** | **BDHEVFP11** | **(-)** |  |  |  |  |  |  |  |  |  |  |  |  |  |  |  |  |  |  |  |  |  |  |  |  | **0** |
| **26** | **BDHEVFP14** | **(+)** |  |  |  |  |  |  |  |  |  |  |  |  |  |  |  |  |  |  |  |  |  |  |  |  | **6** |
| **27** | **BDHEVFP15** | **(+)** |  |  |  |  |  |  |  |  |  |  |  |  |  |  |  |  |  |  |  |  |  |  |  |  | **8** |
| **28** | **BDHEVFP18** | **(+)** |  |  |  |  |  |  |  |  |  |  |  |  |  |  |  |  |  |  |  |  |  |  |  |  | **24** |
| **29** | **BDHEVFP19** | **(-)** |  |  |  |  |  |  |  |  |  |  |  |  |  |  |  |  |  |  |  |  |  |  |  |  | **0** |
| **30** | **BDHEVFP21** | **(-)** |  |  |  |  |  |  |  |  |  |  |  |  |  |  |  |  |  |  |  |  |  |  |  |  | **0** |
| **31** | **BDHEVFP25** | **(+)** |  |  |  |  |  |  |  |  |  |  |  |  |  |  |  |  |  |  |  |  |  |  |  |  | **9** |
| **32** | **BDHEVFP26** | **(-)** |  |  |  |  |  |  |  |  |  |  |  |  |  |  |  |  |  |  |  |  |  |  |  |  | **1** |
| **33** | **BDHEVFP28** | **(+)** |  |  |  |  |  |  |  |  |  |  |  |  |  |  |  |  |  |  |  |  |  |  |  |  | **1** |
| **34** | **BDHEVFP29** | **(-)** |  |  |  |  |  |  |  |  |  |  |  |  |  |  |  |  |  |  |  |  |  |  |  |  | **0** |
| **35** | **BDHEVFP31** | **(+)** |  |  |  |  |  |  |  |  |  |  |  |  |  |  |  |  |  |  |  |  |  |  |  |  | **9** |
| **36** | **BDHEVFP32** | **(-)** |  |  |  |  |  |  |  |  |  |  |  |  |  |  |  |  |  |  |  |  |  |  |  |  | **0** |
| **37** | **BDHEVFP35** | **(-)** |  |  |  |  |  |  |  |  |  |  |  |  |  |  |  |  |  |  |  |  |  |  |  |  | **0** |
| **38** | **BDHEVFP36** | **(+)** |  |  |  |  |  |  |  |  |  |  |  |  |  |  |  |  |  |  |  |  |  |  |  |  | **8** |
| **39** | **BDHEVFP37** | **(+)** |  |  |  |  |  |  |  |  |  |  |  |  |  |  |  |  |  |  |  |  |  |  |  |  | **2** |
| **40** | **BDHEVFP38** | **(+)** |  |  |  |  |  |  |  |  |  |  |  |  |  |  |  |  |  |  |  |  |  |  |  |  | **5** |
| **41** | **BDHEVFP39** | **(+)** |  |  |  |  |  |  |  |  |  |  |  |  |  |  |  |  |  |  |  |  |  |  |  |  | **17** |
| **42** | **BDHEVFP41** | **(+)** |  |  |  |  |  |  |  |  |  |  |  |  |  |  |  |  |  |  |  |  |  |  |  |  | **2** |
| **43** | **BDHEVFP44** | **(+)** |  |  |  |  |  |  |  |  |  |  |  |  |  |  |  |  |  |  |  |  |  |  |  |  | **8** |
| **44** | **BDHEVFP34** | **(-)** |  |  |  |  |  |  |  |  |  |  |  |  |  |  |  |  |  |  |  |  |  |  |  |  | **0** |
| **45** | **BDHEVM144** | **(+)** |  |  |  |  |  |  |  |  |  |  |  |  |  |  |  |  |  |  |  |  |  |  |  |  | **3** |
| **46** | **BDHEVM079** | **(+)** |  |  |  |  |  |  |  |  |  |  |  |  |  |  |  |  |  |  |  |  |  |  |  |  | **24** |
| **47** | **BDHEVM187** | **(+)** |  |  |  |  |  |  |  |  |  |  |  |  |  |  |  |  |  |  |  |  |  |  |  |  | **2** |
| **48** | **BDHEVM162** | **(-)** |  |  |  |  |  |  |  |  |  |  |  |  |  |  |  |  |  |  |  |  |  |  |  |  | **0** |
| **49** | **BDHEVM169** | **(+)** |  |  |  |  |  |  |  |  |  |  |  |  |  |  |  |  |  |  |  |  |  |  |  |  | **16** |
| **50** | **BDHEVM127** | **(+)** |  |  |  |  |  |  |  |  |  |  |  |  |  |  |  |  |  |  |  |  |  |  |  |  | **24** |
| **51** | **BDHEVF120** | **(+)** |  |  |  |  |  |  |  |  |  |  |  |  |  |  |  |  |  |  |  |  |  |  |  |  | **24** |
| **52** | **BDHEVM082** | **(+)** |  |  |  |  |  |  |  |  |  |  |  |  |  |  |  |  |  |  |  |  |  |  |  |  | **24** |
| **53** | **BDHEVM115** | **(+)** |  |  |  |  |  |  |  |  |  |  |  |  |  |  |  |  |  |  |  |  |  |  |  |  | **2** |
| **54** | **BDHEVM033** | **(-)** |  |  |  |  |  |  |  |  |  |  |  |  |  |  |  |  |  |  |  |  |  |  |  |  |  |
| **55** | **BDHEVF090** | **(-)** |  |  |  |  |  |  |  |  |  |  |  |  |  |  |  |  |  |  |  |  |  |  |  |  |  |
| **56** | **BDHEVF170** | **(+)** |  |  |  |  |  |  |  |  |  |  |  |  |  |  |  |  |  |  |  |  |  |  |  |  | **24** |
| **57** | **BDHEVM085** | **(+)** |  |  |  |  |  |  |  |  |  |  |  |  |  |  |  |  |  |  |  |  |  |  |  |  | **24** |
| **58** | **BDHEVM125** | **(+)** |  |  |  |  |  |  |  |  |  |  |  |  |  |  |  |  |  |  |  |  |  |  |  |  | **24** |
| **59** | **BDHEVM077** | **(+)** |  |  |  |  |  |  |  |  |  |  |  |  |  |  |  |  |  |  |  |  |  |  |  |  | **4** |
| **60** | **BDHEVM121** | **(+)** |  |  |  |  |  |  |  |  |  |  |  |  |  |  |  |  |  |  |  |  |  |  |  |  | **2** |
| **61** | **BDHEVM065** | **(+)** |  |  |  |  |  |  |  |  |  |  |  |  |  |  |  |  |  |  |  |  |  |  |  |  | **8** |
| **62** | **BDHEVM085** | **(+)** |  |  |  |  |  |  |  |  |  |  |  |  |  |  |  |  |  |  |  |  |  |  |  |  | **2** |
| **63** | **BDHEVM206** | **(+)** |  |  |  |  |  |  |  |  |  |  |  |  |  |  |  |  |  |  |  |  |  |  |  |  | **8** |
| **64** | **BDHEVMX08** | **(+)** |  |  |  |  |  |  |  |  |  |  |  |  |  |  |  |  |  |  |  |  |  |  |  |  | **24** |
| **65** | **BDHEVM057** | **(-)** |  |  |  |  |  |  |  |  |  |  |  |  |  |  |  |  |  |  |  |  |  |  |  |  | **0** |
| **66** | **BDHEVM134** | **(-)** |  |  |  |  |  |  |  |  |  |  |  |  |  |  |  |  |  |  |  |  |  |  |  |  | **0** |
| **67** | **BDHEVMX02** | **(+)** |  |  |  |  |  |  |  |  |  |  |  |  |  |  |  |  |  |  |  |  |  |  |  |  | **24** |
| **68** | **BDHEVM112** | **(+)** |  |  |  |  |  |  |  |  |  |  |  |  |  |  |  |  |  |  |  |  |  |  |  |  | **24** |
| **69** | **BDHEVM147** | **(-)** |  |  |  |  |  |  |  |  |  |  |  |  |  |  |  |  |  |  |  |  |  |  |  |  | **0** |
| **70** | **BDHEVF105** | **(+)** |  |  |  |  |  |  |  |  |  |  |  |  |  |  |  |  |  |  |  |  |  |  |  |  | **11** |
| **71** | **BDHEVM110** | **(+)** |  |  |  |  |  |  |  |  |  |  |  |  |  |  |  |  |  |  |  |  |  |  |  |  | **4** |
| **72** | **BDHEVM118** | **(+)** |  |  |  |  |  |  |  |  |  |  |  |  |  |  |  |  |  |  |  |  |  |  |  |  | **24** |
| **73** | **BDHEVMX02** | **(+)** |  |  |  |  |  |  |  |  |  |  |  |  |  |  |  |  |  |  |  |  |  |  |  |  | **21** |
| **74** | **BDHEVM089** | **(+)** |  |  |  |  |  |  |  |  |  |  |  |  |  |  |  |  |  |  |  |  |  |  |  |  | **24** |
| **75** | **BDHEVM143** | **(+)** |  |  |  |  |  |  |  |  |  |  |  |  |  |  |  |  |  |  |  |  |  |  |  |  | **3** |
| **76** | **BDHEVM177** | **(+)** |  |  |  |  |  |  |  |  |  |  |  |  |  |  |  |  |  |  |  |  |  |  |  |  | **15** |
| **77** | **BDHEVM128** | **(+)** |  |  |  |  |  |  |  |  |  |  |  |  |  |  |  |  |  |  |  |  |  |  |  |  | **24** |
| **78** | **BDHEVM143** | **(+)** |  |  |  |  |  |  |  |  |  |  |  |  |  |  |  |  |  |  |  |  |  |  |  |  | **24** |
| **79** | **BDHEVF192** | **(+)** |  |  |  |  |  |  |  |  |  |  |  |  |  |  |  |  |  |  |  |  |  |  |  |  | **12** |
| **80** | **BDHEVF055** | **(+)** |  |  |  |  |  |  |  |  |  |  |  |  |  |  |  |  |  |  |  |  |  |  |  |  | **8** |
| **81** | **BDHEVF155** | **(+)** |  |  |  |  |  |  |  |  |  |  |  |  |  |  |  |  |  |  |  |  |  |  |  |  | **2** |
| **82** | **BDHEVM066** | **(+)** |  |  |  |  |  |  |  |  |  |  |  |  |  |  |  |  |  |  |  |  |  |  |  |  | **2** |
| **83** | **BDHEVF126** | **(+)** |  |  |  |  |  |  |  |  |  |  |  |  |  |  |  |  |  |  |  |  |  |  |  |  | **24** |
| **84** | **BDHEVM129** | **(-)** |  |  |  |  |  |  |  |  |  |  |  |  |  |  |  |  |  |  |  |  |  |  |  |  | **0** |
| **85** | **BDHEVM067** | **(+)** |  |  |  |  |  |  |  |  |  |  |  |  |  |  |  |  |  |  |  |  |  |  |  |  | **3** |
| **86** | **BDHEVM143** | **(+)** |  |  |  |  |  |  |  |  |  |  |  |  |  |  |  |  |  |  |  |  |  |  |  |  | **20** |
| **87** | **BDHEVM051** | **(+)** |  |  |  |  |  |  |  |  |  |  |  |  |  |  |  |  |  |  |  |  |  |  |  |  | **2** |
| **88** | **BDHEVM188** | **(+)** |  |  |  |  |  |  |  |  |  |  |  |  |  |  |  |  |  |  |  |  |  |  |  |  | **24** |
| **89** | **BDHEVM198** | **(+)** |  |  |  |  |  |  |  |  |  |  |  |  |  |  |  |  |  |  |  |  |  |  |  |  | **24** |
| **90** | **BDHEVF151** | **(+)** |  |  |  |  |  |  |  |  |  |  |  |  |  |  |  |  |  |  |  |  |  |  |  |  | **24** |
| **91** | **BDHEVM053** | **(+)** |  |  |  |  |  |  |  |  |  |  |  |  |  |  |  |  |  |  |  |  |  |  |  |  | **2** |
| **92** | **BDHEVM213** | **(+)** |  |  |  |  |  |  |  |  |  |  |  |  |  |  |  |  |  |  |  |  |  |  |  |  | **2** |
