# Supplementary figures and images for: A tightly clustered hepatitis E virus genotype 1a is associated with endemic and outbreak infections in Bangladesh
Source: PLoS One. 2021 Jul 22;16(7):e0255054. doi: 10.1371/journal.pone.0255054 (PMC8297744; doi:10.1371/journal.pone.0255054)

S4 File


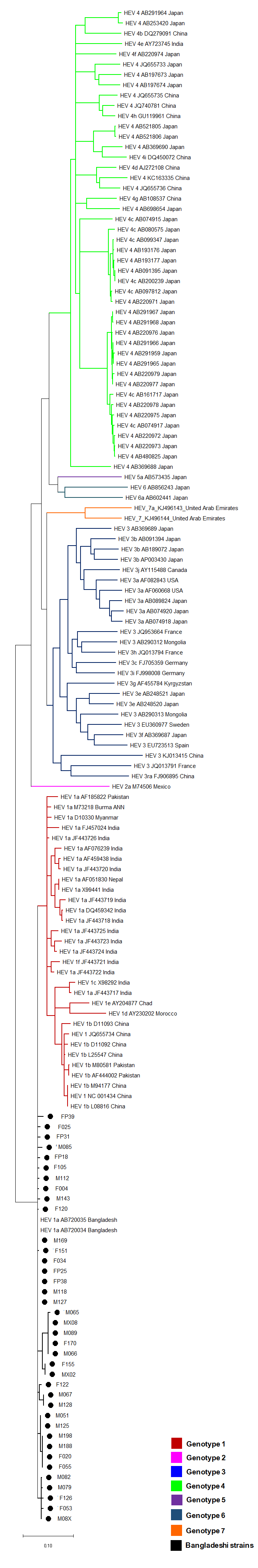

Supplement: S5 File — A midpoint rooted tree showing the relationship between the 38 Bangladeshi HEV sequences with 109 reference sequences representing all genotype and subtypes. The tree was constructed using RAxML v7.2.8 available in Geneious software using GTR+G+I nucleotide substitution model with 500 bootstrapping replicates. The Bangladeshi HEV strains from this study is presented as ● followed by strain number, and the reference genomes are presented as genotype, subtype followed by GenBank accession number and the country of origin. The scale bar indicates the number of nucleotide substitution. (DOCX) [file pone.0255054.s005.docx]
