# Supplementary material for: A tightly clustered hepatitis E virus genotype 1a is associated with endemic and outbreak infections in Bangladesh
Source: PLoS One. 2021 Jul 22;16(7):e0255054. doi: 10.1371/journal.pone.0255054 (PMC8297744; doi:10.1371/journal.pone.0255054)
Supplement: Attachment — Submitted filename: Response to reviewers.docx [file pone.0255054.s006.docx]

**Response to reviewer’s:**

PONE-D-20-39475
A tightly clustered hepatitis E virus genotype 1a is associated with endemic and outbreak infections in Bangladesh
PLOS ONE

Dear Dr. Rahman,

Thank you for submitting your manuscript to PLOS ONE. After careful consideration, we feel that it has merit but does not fully meet PLOS ONE’s publication criteria as it currently stands. Therefore, we invite you to submit a revised version of the manuscript that addresses the points raised during the review process.

Please provide the details requested by the reviewer and explain the process of the full sequence validation using RACE PCR. As noted the abstract line 40 is confusing as we may understand that 38+21 strains were typed while there were 38 genotyped strains from which 21 full sequences were obtained.

**Response: We have rephrased the sentence and warding to address the confusion on the number of strains genotyped and the number of strains with whole genome sequenced. Plz see line 38-42.**

Please submit your revised manuscript by Apr 10 2021 11:59PM. If you will need more time than this to complete your revisions, please reply to this message or contact the journal office at [plosone@plos.org](mailto:plosone@plos.org). When you're ready to submit your revision, log on to <https://www.editorialmanager.com/pone/> and select the 'Submissions Needing Revision' folder to locate your manuscript file.

Please include the following items when submitting your revised manuscript:

- A rebuttal letter that responds to each point raised by the academic editor and reviewer(s). You should upload this letter as a separate file labeled 'Response to Reviewers'.

**Response: We have uploaded separate file as “Response to Reviewers”.**

- A marked-up copy of your manuscript that highlights changes made to the original version. You should upload this as a separate file labeled 'Revised Manuscript with Track Changes'.

**Response: We have uploaded a file labeled “Revised Manuscript with Track Change”.**

- An unmarked version of your revised paper without tracked changes. You should upload this as a separate file labeled 'Manuscript'.

**Response: We have uploaded a file labeled “Manuscript”.**

If you would like to make changes to your financial disclosure, please include your updated statement in your cover letter. Guidelines for resubmitting your figure files are available below the reviewer comments at the end of this letter.

**Response: We have included the updated financial disclosure statement in Cover letter.**

If applicable, we recommend that you deposit your laboratory protocols in protocols.io to enhance the reproducibility of your results. Protocols.io assigns your protocol its own identifier (DOI) so that it can be cited independently in the future. For instructions see: <http://journals.plos.org/plosone/s/submission-guidelines#loc-laboratory-protocols>

We look forward to receiving your revised manuscript.

Kind regards,

Pierre Roques, Ph.D.
Academic Editor
PLOS ONE

Journal Requirements:

When submitting your revision, we need you to address these additional requirements.

1. Please ensure that your manuscript meets PLOS ONE's style requirements, including those for file naming. The PLOS ONE style templates can be found at

<https://journals.plos.org/plosone/s/file?id=wjVg/PLOSOne_formatting_sample_main_body.pdf> and

[https://journals.plos.org/plosone/s/file?id=ba62/PLOSOne_formatting_sample_title_authors_affiliations.pdf](about:blank)

**Response: We have followed the guidelines**

2. As part of your revisions please provide additional information about the source/origin of the samples utilized for this study. You indicate in your Methods that you collected samples that had been submitted to the virology department of BSMMU and that these samples had been systematically selected, i.e.  Systematically first four HEV IgM ELISA positive patients each month.

Please indicate whether the patients or next of kin provided informed consent for the use of these samples and the form of consent (written or oral). If the samples were anonymized please state so.

**Response: We have revised the section as suggested by the reviewer and mentioned the how samples were anonymized. Plz see line 124-129.**

Thank you for your attention in this matter.

3. Thank you for stating the following in the Acknowledgments Section of your manuscript:

'This study was supported by the Oxford University Clinical Research Unit, Vietnam.'

We note that you have provided funding information that is not currently declared in your Funding Statement. However, funding information should not appear in the Acknowledgments section or other areas of your manuscript. We will only publish funding information present in the Funding Statement section of the online submission form.

a. Please remove any funding-related text from the manuscript and let us know how you would like to update your Funding Statement. Currently, your Funding Statement reads as follows:

'The author(s) received no specific funding for this work.'

b. Please include your amended statements within your cover letter; we will change the online submission form on your behalf.

**Response: We have removed the funding information from acknowledgement section. We have added the amended information in the revised cover letter. We have added new text in acknowledgement section.**

Additional Editor Comments:

sorry for the delay in the reviewing process.

Please review your reference list to ensure that it is complete and correct. If you have cited papers that have been retracted, please include the rationale for doing so in the manuscript text, or remove these references and replace them with relevant current references. Any changes to the reference list should be mentioned in the rebuttal letter that accompanies your revised manuscript. If you need to cite a retracted article, indicate the article’s retracted status in the References list and also include a citation and full reference for the retraction notice.

**Response: We have reviewed the references and couldn’t find any retracted article.**

[Note: HTML markup is below. Please do not edit.]

Reviewers' comments:

Reviewer's Responses to Questions

**Comments to the Author**

1. Is the manuscript technically sound, and do the data support the conclusions?

The manuscript must describe a technically sound piece of scientific research with data that supports the conclusions. Experiments must have been conducted rigorously, with appropriate controls, replication, and sample sizes. The conclusions must be drawn appropriately based on the data presented.

Reviewer #1: Yes

2. Has the statistical analysis been performed appropriately and rigorously?

Reviewer #1: I Don't Know

3. Have the authors made all data underlying the findings in their manuscript fully available?

The [PLOS Data policy](http://www.plosone.org/static/policies.action#sharing) requires authors to make all data underlying the findings described in their manuscript fully available without restriction, with rare exception (please refer to the Data Availability Statement in the manuscript PDF file). The data should be provided as part of the manuscript or its supporting information, or deposited to a public repository. For example, in addition to summary statistics, the data points behind means, medians and variance measures should be available. If there are restrictions on publicly sharing data—e.g. participant privacy or use of data from a third party—those must be specified.

Reviewer #1: Yes

4. Is the manuscript presented in an intelligible fashion and written in standard English?

PLOS ONE does not copyedit accepted manuscripts, so the language in submitted articles must be clear, correct, and unambiguous. Any typographical or grammatical errors should be corrected at revision, so please note any specific errors here.

Reviewer #1: Yes

5. Review Comments to the Author

Please use the space provided to explain your answers to the questions above. You may also include additional comments for the author, including concerns about dual publication, research ethics, or publication ethics. (Please upload your review as an attachment if it exceeds 20,000 characters)

Reviewer #1: Hao and colleagues describe the closely grouped hepatitis E virus genotype 1a associated with endemic and outbreak infections in Bangladesh. The paper is quite interesting, I have some concerns.

Line 40 ; Lines 43-44 ; lines-239-240 : Authors should provide precisions at this level for a better understanding. Is it the 21 sequences from complete genome sequence or the 38 sequences from incomplete genome sequence.

**Response: We have rephrased the sentence as following; “A 279 bp open reading frame (ORF) 2 and ORF 3 sequence was obtained from 54.2% (38/70) of the strains. Of these, whole genome sequence (WGS) was obtained from 21 strains. In phylogenetic analysis using the 279 bp sequence all HEV sequences belonged to genotype 1 and subtype 1a” . Plz see Abstract line 38 to 42.**

Lines 121-124 : The authors should provide more details on the selection criteria for the first four ELISA-positive patients of the month, in order to better interpret the results, e.g., did these patients have any symptoms of the disease or not ?

**Response: We agree with the reviewer that clinical information of the patients would have been helpful to interpret the data. However, this component of the study was a genome surveillance for HEV to understand the genomic epidemiology and genotype of HEV strains circulating in Bangladesh. Patients with HEV IgM test request and HEV IgM positive were eligible for including in the study. We selected the first four HEV IgM positive samples each month. Samples were unlinked with patient data be replacing the patient ID with a study ID. We had limited patient information e.g. age, gender and pregnancy status (in case of female) of the patients. This has been mentioned in the limitations of the study. We have revised study design and population section. Plz see line 122 to 129 and line 343.**

Line 129 : Review the title of the section, it's « Amplification and sequencing »

**Response: We have revised the title of the section.**

Lines 134-137 : What was the point of doing this RACE PCR, given that only the 21 sequences underwent a phylogenetic analysis?

**Response: We apologies for the confusion. RACE PCR was not performed for all strains. In strains where maximum number of overlapping fragments could be amplified and sequenced a RACE PCR was conducted to complete the 5’ and 3’ region. Number of fragments amplified and sequenced for each strain are presented “S File 4; fragment map”. We have also revised the text; Plz see line 140-142**

Lines 143-144 : What is the point to aligne overlapping sequences?

**Response: For each strain we tried to amplify and sequence the whole genome in 24 fragments. Overlapping sequences were aligned to generate the full length sequences where all fragments could be amplified and sequenced (n=21). The number of fragments amplified and sequenced are presented in “S File 4; fragment map”. We have revised the warding to clarify it. Plz see line 149-150**

Lines 194-195 : The authors report that at least one DNA fragment could be sequenced in all plasma samples. However, only 38 sequences ((17 from incomplete genome sequence and 21 from complete genome sequence) on the 70 PCR positive samples could be obtained and sequenced.

**Response: We apologies for the confusion. We attempted to sequence complete genome of all strains (n=70), however we could not amplify and sequence all 24 fragments for each stains. Fragments amplified and sequenced for each strain is presented “S file 4”. We could amplify 825 fragments from 70 strains. Of these, all 24 fragments were amplified and sequenced for 21 strains. All 24 fragments could not be amplified or sequenced for the remaining 49 strains. Of these strains (n=49), along with other fragments, the 279 bp ORF2 and ORF 3 region (Fragment no 20 (S20)) could be amplified and sequenced for 17 strains.**

Lines 197-198 : How do the authors explain this difference with the sequences obtained by WGS (21 complete genome sequences) and RACE PCR (38 incomplete genome sequences) ?

**Response: RACE PCR was not conducted for strains with incomplete genome sequences. We conducted RACE PCR for 21 stains (21 complete genome sequence) to complete the 5’ and 3’ ends. We have revised the text on this, plz see line 140-142**

Lines 210-126 : Is this high sequence conservation observed only at the ORF1 sequences level ?

**Response: We observed high sequence conservation in all three region among isolates where WGS were available.**

Lines 275-276 : Aren't the two arguments reported by the authors linked?

**Response: We have revised the arguments. Plz see line 285.**

Lines 276-282 : These explanations of the authors are in contradiction with those reported at the level of the results (lines 189-190).

**Response: We apologies for the confusion. In Line 189-190, we compared all patients (both male and female) recruited during 2013-14 and 2014-15. In line 276-282, we compared difference among male and female patients. We have rephrased the sentence and added data on RNA positivity among male and female patients in results section. Plz see line 194-200.**

Lines 283-285 : How do the authors explain this distinction of the Bangladeshi HEV strains within the genotype 1a cluster ?

**Response: The exact reason for this clustering among HEV genotype 1a strains is unknown, but it is possible that viral evolution and host genetic adaption of these viruses in Bangladeshi population might be an underlying reason. We have included a comment in the discussion, plz see line 302-303**

Lines 290-292 : Doesn't this explanation contradict the argument that the observed discrepancy between IgM and RNA-positive levels is due to a resolution of the active infection ?

**Response: We agree with the reviewer and removed the sentence: i) active HEV infection was resolved when the patient visited the hospital. Plz see line 285.**

Line 318 : Can it be said that strains of genotype 1a are dominant in Bangladesh, since the authors themselves reported that their study had several limitations

**Response: we have made the suggestive change. Plz see line 328**

6. PLOS authors have the option to publish the peer review history of their article ([what does this mean?](https://journals.plos.org/plosone/s/editorial-and-peer-review-process#loc-peer-review-history)). If published, this will include your full peer review and any attached files.


If you choose “no”, your identity will remain anonymous but your review may still be made public.


**Do you want your identity to be public for this peer review?** For information about this choice, including consent withdrawal, please see our [Privacy Policy](https://www.plos.org/privacy-policy).

Reviewer #1: No

[NOTE: If reviewer comments were submitted as an attachment file, they will be attached to this email and accessible via the submission site. Please log into your account, locate the manuscript record, and check for the action link "View Attachments". If this link does not appear, there are no attachment files.]

While revising your submission, please upload your figure files to the Preflight Analysis and Conversion Engine (PACE) digital diagnostic tool, <https://pacev2.apexcovantage.com/>. PACE helps ensure that figures meet PLOS requirements. To use PACE, you must first register as a user. Registration is free. Then, login and navigate to the UPLOAD tab, where you will find detailed instructions on how to use the tool. If you encounter any issues or have any questions when using PACE, please email PLOS at [figures@plos.org](mailto:figures@plos.org). Please note that Supporting Information files do not need this step.

**Response: We have uploaded and the figures in PACE and the figures meet PLOS requirements**
