# Supplementary material for: A tightly clustered hepatitis E virus genotype 1a is associated with endemic and outbreak infections in Bangladesh
Source: PLoS One. 2021 Jul 22;16(7):e0255054. doi: 10.1371/journal.pone.0255054 (PMC8297744; doi:10.1371/journal.pone.0255054)
Supplement: Attachment — Submitted filename: Response to reviewer.docx [file pone.0255054.s007.docx]

6. Review Comments to the Author

Please use the space provided to explain your answers to the questions above. You may also include additional comments for the author, including concerns about dual publication, research ethics, or publication ethics. (Please upload your review as an attachment if it exceeds 20,000 characters)

Reviewer #1: Some of my concerns about the study still remain.
Line 31: Authors should modify "HEV infected patients" to HEV immunoglobulin M (IgM) positive patients

Response: We have modified the sentence as suggested, plz see line 31

Lines 40-42: The authors should clarify, because it seemed to me that the phylogenetic analysis was performed with the 21 WGS sequences of HEV (Figure 1 and 2)

Response: We have modified the sentence to clarify it, plz see line 41-44

Line 132: In this section, what was the methodology used to obtain the 38 sequences of 279 bp.

Response: We apologize for the confusion. The 279 bp fragment was amplified and sequenced by primer set 20 (S1 File). We have added clarification, plz see line 145-147

Line 137: To Clarify the nested PCR method used: What primers were used? What were the expected fragment sizes?

Response: For nested PCR we used 20 sets of primers (each set contains 4 primers (two external and two internal). Plz see details in S 1 File. The fragment size was 450-600 bp, plz see line 139-140.

Lines 167-168: The authors must specify that it is on the 21 HEV WGS that the mutations have been identified.

Response: We have specified as suggested. Plz see line 172-173

Lines 225-227: these data are not available in the figures (Fig 1 and 2).

Response: We agree with the reviewer. We have added a supplementary figure (S4 File) to present the data.

Lines 264-267: The authors should note that the analysis of virulence-associated mutations was limited to the 21 WGS (Table 1)

Response: We have mentioned in the title of Table 1. Plz see table 1 and table 2

Lines 300-302: Molecular detection tests (qPCR) by reference are more sensitive than serological tests (Ig anti-HEV). Thus, the difference observed in this study is not rather related to the non-existence of a serological test of reference.

Response: We have modified the sentence to address the issue.

Lines302-307: The authors must specify that this observed difference was not significant (P=0.08)

Response: We have revised the sentence.
