# Supplementary material for: A tightly clustered hepatitis E virus genotype 1a is associated with endemic and outbreak infections in Bangladesh
Source: PLoS One. 2021 Jul 22;16(7):e0255054. doi: 10.1371/journal.pone.0255054 (PMC8297744; doi:10.1371/journal.pone.0255054)
Supplement: Attachment — Submitted filename: Response to reviewer_270521.docx [file pone.0255054.s008.docx]

**Response to Reviewer:**

Please correct the minor points raised by the reviewer and correct the supplement table 1 with the primers as the indication of sense and antisense are misleading and confusing.

In addition, numbering of the reverse-sense primers should be also reversed as an example

| HEV-S2-A1 | 1st antisense | **GTTCAGCGYTGGTATACTGC** | 348 - 367 |
| --- | --- | --- | --- |

should be numbered as 367 - 348 to  indicate that the sequence is read on the negative (-) strand ; in extenso in the 3' to 5' direction on the positive (+) strand that is the reference sequence used to number the position.

In this S1 file all primers' sets are for nested PCR and named it 1st sense, 2nd sense then 1st antisense, 2nd antisense but the numbering you indicated clearly shown that the order is 1st sense, 1st antisense, 2nd sense, 2nd antisense.

**Response**

We have revised the S1 File and corrected the antisense primer position numbering. The primer sequence is already reverse. We have included the the reference sequence used to number the positions.

We have corrected the organization of primers as 1st sense, 1st antisense, 2nd sense, 2nd antisense.

Please clarify and explain the size of the Set 20 product (i.e how you read 279 base pairs at the end).

**Response:**

The fragment 20 will amplify a `440bp fragment. Of these 440 bp fragment, we extracted 279 bp for genotyping (Ref 26). We have revised the sentence as follows and added a new reference. “We have revised the sentence for clarification “The entire genome sequences (amplified by primer set 1 to 20 (S1 File)) and a 279bp region (position 5972-6319 of GenBank accession no M73218) of ORF2 and ORF3 (part of the `440 bp fragment amplified by primer set 20 (S1 File)) were used for final analysis [26]” plz see page 145-148.
